# Personal network protects, social media harms: Evidence from two surveys during the COVID-19 pandemic

**DOI:** 10.3389/fpsyg.2022.964994

**Published:** 2022-08-22

**Authors:** Ruqin Ren, Bei Yan

**Affiliations:** ^1^Institute of Cultural and Creative Industry, Shanghai Jiao Tong University, Shanghai, China; ^2^School of Business, Stevens Institute of Technology, Hoboken, NJ, United States

**Keywords:** anxiety, social networks, social media use, news exposure, COVID-19

## Abstract

**Background:**

The classic debate regarding the complex relationships between personal network, social media use, and mental well-being requires renewed examination in the novel context of pandemic-related social isolation.

**Data and method:**

We present two surveys conducted at (i) the earlier months of the pandemic and (ii) the end of large scale social-lockdown measures in the U.S. to explore the social and behavioral antecedents of mental health states relating to social media use. Study 1 tracked the longitudinal changes of personal network, social media use, and anxiety level of a group of individuals (*N* = 147) over a three-month period during the pandemic. Study 2 replicated and extended the theoretical model to a race-representative U.S. adult sample (*N* = 258).

**Results:**

Both studies consistently show that (1) more time on social media worsens anxiety. It also mediates the relationship between personal network size and anxiety. That is, a small personal network predicts more social media use, which is in turn related to increased anxiety. (2) Moreover, the effect of social media use on anxiety is mainly explained by news consumption on social media, rather than non-news related usage. (3) This link’s strength is moderated by one’s perception of COVID-19 impact, such that news consumption on social media increases anxiety more when the perceived impact is higher.

**Conclusion:**

These results demonstrate communication technologies’ increasingly critical and multifaceted role in affecting mental health conditions.

## Introduction

Associated with the rapid spread of the COVID-19 pandemic is the escalated mental health concerns for the public (Pfefferbaum and North, 2020). We are not yet clear about the acute or long-term consequences of the COVID-19 lockdown measures on vulnerable groups (Cauberghe et al., 2021). This study explores the mental health implications of social isolation and examines whether social media is helpful or detrimental to people secluded during the pandemic.

The relationships between social media, sociality, and mental well-being have been a topic of debate for decades, but the existing findings are mixed (Twenge et al., 2020). The “digital media harm” view argued that social media use is associated with worse mental health conditions (Orben and Przybylski, 2019). The more optimistic view suggested that social media connects us by providing an accessible or enhanced channel to find new contacts, maintain existing relationships and join online communities; thus, it could be beneficial for mental health (Bessière et al., 2008).

COVID-19 further complicated the already complex link between personal network, social media, and well-being. We are thus motivated by these new characteristics that may challenge the generalizability of existing findings. First, as social distancing limited much face-to-face communication and gatherings, social media has become a critical way to satisfy social interaction needs. When used properly, social media may serve as a constructive coping strategy that can reduce anxious feelings during the COVID-19 quarantine (Cauberghe et al., 2021). Second, increased social media use has been associated with several negative mental outcomes (Orben and Przybylski, 2019). The COVID-induced reliance on social media (Valdez et al., 2020) may exacerbate these negative effects and result in an intensified feeling of loneliness, fear, and anxiety. Third, a sense of isolation and loneliness may be more widely shared in a time of pandemic and social distancing. This changing nature of “isolation” will inevitably change patterns of social media use. It is thus both theoretically important and practically relevant to continue the investigation about the impact of social media use on mental health in this context.

Joining a stream of research that has extensively documented the relationships between social media use and mental well-being, we extend prior research in three aspects. First, we conducted two studies using samples of distinct demographic characteristics. Study 1 is a longitudinal survey that tracks social media usage and anxiety levels of a group of individuals over three months in the earlier stage of the pandemic. Study 2 was conducted at the end of May 2021, when the U.S. gradually resumes normal life. It contains a race-representative sample of U.S. adults to generalize and extend the findings obtained in Study 1. Second, it introduces one’s personal network as an important social antecedent of social media usage. Third, the present study distinguishes between social media use for different purposes and proposes a conditional effect (moderated mediation) of one’s perception of COVID-19 impact. This will help better determine “why” and “for whom” social media use matters.

### Personal network and mental health

A large body of social and epidemiologic research has established social support as a critical predictor of improved physical and psychiatric conditions (Child and Lawton, 2020). One aspect of social support that is often the focus of social network research is the size of one’s personal network (Marin and Hampton, 2007). This type of network is made up of a focal person’s nominated social contacts in a name generator question (Child and Lawton, 2020). Having a large personal network indicates a high level of perceived social support.

Being embedded in strong social networks can improve mental health by activating several social-psychological mechanisms. The social influence mechanism encourages individuals to conform to the normative behaviors popular in one’s social circle. A survey during COVID-19 showed that people adhered to social distancing measures most when they thought their close social circle did (Tunçgenç et al., 2021). The social resource mechanism explains how one gains emotional, instrumental, informational and appraisal support from their social contacts (Kanekar and Sharma, 2020; Yamamoto et al., 2020). In addition, the *stress-buffering* model works by modulating responses to negative life events that may cause mental illness. A longitudinal study of Swiss elders confirmed that self-reported satisfaction with communication during the COVID-19 was associated with less decline in positive affect and less increase in loneliness (Macdonald and Hülür, 2021). Thus, we re-test this protective effect of personal network:

**RQ1:** Does personal network size decrease anxiety level?

### Personal network and social media

Literature about the link between one’s social network and social media use can be broadly split into two camps. A first camp assumes that maintaining social relationships and social media usage are somewhat at odds, and thus argues that there may be a *trade-off* effect between size of personal network and social media use (Bessière et al., 2008). Two theoretical reasons can explain why more social connections predict less time spent on social media.

First, the *time constraint* mechanism argues that maintaining social relations requires time and energy (Dunbar, 2016), so having more offline social connections will reduce time spent on social media. An assumption of their argument is that social media is not useful for maintaining strong ties and people spend most of their social media time on asocial activities. Also, online networks may not be as useful in providing social support compared to offline networks (Mazzoni et al., 2016). A critique of these assumptions is that social media today serves a wide range of purposes, which earlier studies did not assess. In a pandemic, it is difficult to determine if social media use is a “solitary” activity or “social” activity. Second, personal predisposing characteristics precede social media use – that is, isolated or lonely feelings predict more addictive internet use (Brand et al., 2019). Individuals who are not well connected socially may need to satisfy the unmet social needs, find entertainment, or escape daily life *via* social media. Empirical findings confirmed that people use social media more when they have small social networks (Hill and Zheng, 2018; Boursier et al., 2020) or feel lonely (Cauberghe et al., 2021). In the pandemic, this motivation may be particularly strong because the average anxiety and depression level increased for most people (Taylor et al., 2020). Both mechanisms would thus predict that small personal networks increase social media use (the *trade-off* effect).

Nonetheless, another camp predicts an opposite and positive relationship between personal network and social media use (the *social augmentation* effect). Social media is considered as an extension of one’s sociality. People who excel at offline social interactions were found to be also good at developing online relationships (Vergeer and Pelzer, 2009; Rosenfeld and Thomas, 2012). Social media also lend themselves well to socializing with new people with diverse backgrounds and revitalizing old connections (Dunbar, 2016). Thus, having a large network may be positively related to spending more time on social media.

Due to the theoretical inconsistency between the *trade-off* and the *social augmentation* effect, we ask:

**RQ2**: What is the relationship between personal network and time spent on social media use?

### Social media use and anxiety

The paradoxical relationship between social media use and mental health has been a topic of extensive debate since the 1990s. Two contradictory predictions have been discussed. The negative perspective states that frequent use of the Internet harms mental health outcomes (Orben and Przybylski, 2019). Possible theoretical explanations suggested so far include (1) the limited capacity theory: maintaining online social networks erodes time and cognitive energy that could have been spent with offline and truly meaningful connection (Dunbar, 2016); (2) the friendship paradox and the happiness paradox: in a friendship network, it can be mathematically proven that my friends are more popular and happier than me on average (Bollen et al., 2017); (3) the contagious emotion theory: during times of crisis, the contagious and negative sentiments will quickly saturate online communities with the help of social media which further leads to collective mental challenges (Iglesias-Sánchez et al., 2020; Valdez et al., 2020). The positive perspective, however, argues that mental well-being improves by receiving social support, regardless of the communication channel used. Increased social media use thus substitutes for what can be established in offline interactions and provides even more accessible ways of communication (Bessière et al., 2008).

In the context of COVID-related quarantine, both perspectives have received some empirical support. On the negative side, cross-sectional survey studies of Chinese adults show that a higher amount of social media exposure was positively associated with higher odds of anxiety (Gao et al., 2020) and more negative affect and secondary traumatic stress (Zhao and Zhou, 2020). Similarly, a cross-sectional survey with participants from four countries (Geirdal et al., 2021) and an Italy-based cross-sectional study during the lockdown (Boursier et al., 2020) reported that longer social media use was associated with significantly poorer mental health conditions.

Meanwhile, some other scholars noted the positive side of social media as a coping mechanism to reduce information uncertainty and anxiety. A cross-sectional survey among Belgian adolescents revealed that actively monitoring the COVID-19 situation and trying to learn more about preventive measures *via* social media is useful for boosting feelings of happiness (Cauberghe et al., 2021). A social media content analysis using a Spanish corpus explored the contagious emotions present on social media and discussed how this might be an opportunity to use it as a “collective therapy” to allow for positive affect to spread (Iglesias-Sánchez et al., 2020). Social media also provided a critical channel for users to proactively seek health information, such as knowledge about vaccines and preventive measures, which may be helpful in reducing the perceived risks associated with the disease (Li and Zheng, 2022; Zheng et al., 2022). These studies, however, were unable to ascertain the causal direction between social media use and mental conditions. Arguably, social media could be a compensatory tool to satisfy unmet social or informational needs (Mazzoni et al., 2016; Brand et al., 2019).

Considering the mixed findings so far, we ask the following research question:

**RQ3**: What is the relationship between time spent on social media use and anxiety?

### Differentiating social media use for news and non-news purposes

Many media psychology studies conducted before the pandemic have found that different types of social media use have differential effects on mental health outcomes (Kingsbury et al., 2021). Most of these studies have not yet produced consistent findings. For example, Frison and Eggermont (2016) distinguished between three types of Facebook use (active public, active private, and passive) to examine their differential associations with depression among a sample of Belgium adolescents. Passive and active public use was found to predict depressed mood, but active private use was not. A cross-sectional study among Norwegian university students assessed five social media use types (passive social, passive non-social, active non-social, active social public, and active social private use) and their nuanced associations with suicide intentions (Kingsbury et al., 2021). Non-social use of social media was associated with decreased suicide intentions. Whereas the empirical findings remain mixed, one message is consistent: differential social media use types lead to differential mental effects and the mechanisms are highly complicated (Sharma et al., 2020).

Our research focuses on two types of common social media use: news-related versus non-news-related. Whereas social media is commonly used for non-news purposes such as sharing one’s life and socializing with one’s personal network (Bessière et al., 2010), recent studies on COVID-19 particularly emphasized the “information seeking” affordance of the technology. Using social media to access news has long been established as a key social media usage type (Glynn et al., 2012). In a highly risky and uncertain situation like COVID-19, obtaining news online becomes necessary for many. Digital trace data revealed that both the overall social media usage (Valdez et al., 2020) and Google search queries (Bento et al., 2020; Galido et al., 2021) soared during the early months of the pandemic. Some researchers even argued that users are not as keen with the “social” features of social media, and information retrieval becomes the primary purpose (Kaya, 2020).

News-related social media use has been found to be particularly relevant to mental health during the pandemic. While the increased reliance on social media for news may be used to combat feelings of uncertainty, it may actually result in worsened mental health outcomes (Aalbers et al., 2019). A pre-COVID study found that lower satisfaction with one’s life was significantly associated with increased Facebook news usage (Glynn et al., 2012). During the pandemic, an Iran-based cross-sectional survey found that people who followed more COVID-related news tend to experience higher anxiety (Moghanibashi-Mansourieh, 2020). A Chinese online survey showed that spending ≥2 h daily on COVID-19 news *via* social media were associated with probable anxiety and probable depression in adults (Ni et al., 2020). Contrary to these studies reporting the negative impact of news use, an online survey conducted in Cyprus during the lockdown revealed that users’ social media news use during COVID-19 did not create panic and did not affect the well-being of users (Kaya, 2020).

However, the above studies were conducted during earlier months of the pandemic, and the heightened interests in accessing COVID-related information appeared to be quite short-lived—people’s search interests triggered by local COVID cases did not last for longer than 2 weeks (Bento et al., 2020). The studies were mostly cross-sectional and produced mixed findings regarding the link between news consumption on social media and mental health. We thus follow these studies in distinguishing between social media use for news versus non-news purposes and explore these effects with a longitudinal research design. This distinction will help shed light on *why* social media benefits (or harms) and clarify the boundary conditions of this impact. This leads to a research question:

**RQ4**: Do social media use for news-related vs. non-news purposes have differential effects on anxiety?

### The role of perceived COVID-19 impact

The perceived impact of COVID-19 is an important variable significantly associated with a battery of important social and health outcomes, including health anxiety, financial worry, loneliness, perceived social support (Tull et al., 2020), and psychological distress (H. Wang et al., 2020). Of particular interest to our study, perceived severity of COVID-19 matters for one’s health information behavior. A U.S.-based online survey found that perceived COVID-19 severity and perceived susceptibility to infection are predictors of one’s information seeking behaviors (Qu et al., 2021). When the perceived severity is high, people probably become more sensitive to the uncomfortable feeling associated with news consumption. Information avoidance becomes a strategy to cope with the stress and frustration brought by news.

However, no study yet has addressed if one’s subjective perception of COVID-19 conditions the strength of the link between social media news use and anxiety. It is possible to speculate that those who do not perceive the situation to be severe would not be as influenced psychologically when they were exposed to COVID-related news on social media. They might just consider the news to be irrelevant. However, when people who felt negatively impacted consume lots of news on social media, it may further exacerbate the negative effect of social media news on anxiety (Boursier et al., 2020; Stainback et al., 2020). We thus are also interested in understanding the moderating effect of perceived COVID-19 impact, or whether those who perceive more impact of COVID-19 will be hurt more by the negative impact of social media news use.

**RQ5**: Does perceived COVID-19 impact moderate the relationship between social media news use and anxiety? If yes, how?

### Summary of research objectives

Taken together, the goal of the current research is to explicate the relationship between personal network, social media use and mental health during COVID-19. We report two studies to answer our proposed research questions. The first study aims to examine the effect of social media use on anxiety. It also probes an antecedent of social media use—personal network size. Moreover, Study 1 tests whether the impact of social media differs by its usage type. The objective of the second study is two-fold: 1) to replicate Study 1 using data from a different subpopulation; and 2) to examine the effect of a moderator—perceived COVID-19 impact—on the effect of social media use on mental health.

## Materials and methods

We test the proposed models with two surveys that examine (i) a subpopulation suffering from COVID-related discrimination, and (ii) a cross-sectional, race-representative U.S. sample.

### Study 1: Data collection

This study recruited a sample of East Asian international students pursuing a degree in the U.S. higher education system. East Asian students are noteworthy for this study, especially at the earlier months of the pandemic. The group simultaneously suffer from at least four layers of stressors: (a) The fear of virus infection and the pandemic-related social isolation experienced by the general public; (b) The stressful and difficult adaptation experiences (Jang, 2016); (c) Anti-Asian discourse or even behaviors due to the controversy about the East Asian origin of the coronavirus (Gover et al., 2020; Litam, 2020); (d) Adaptation challenges unique to the East Asian culture (Liu, 2009). We are interested in exploring whether digital communication tools can help alleviate the negative affect experienced by the group.

We conducted a two-wave survey spanning 3 months. From May 10 to May 15, 2020, we sent out the first wave of the web-based survey to a nationwide sample of East Asian international students. The sample is drawn from a wide variety of sources, including online forums of international students, online forums for East Asian immigrants in general (with a special focus on Chinese, Korean and Japanese immigrants), campus-specific Facebook groups of international student associations across the U.S., and instant messenger groups created by international students. After removing one participant that completed the questionnaire under a minimum necessary time of 2 min (this threshold is set as the minimum time needed to read through and process all the questions based on our pilot tests), the first wave received 251 completed responses. From August 15 to August 20, 2020, 3 months after the initial survey, we followed up with the second wave. This wave returned 149 complete responses (59.6% follow up rate). We then excluded (1) one person who reported having used social media for an unrealistic amount of time—over 20 h a day in Wave 1, and (2) one person who self-reported to be 17-year-old, although all respondents confirmed themselves to be above 18 in the consent form before starting the survey. The following analyses were from this final set of 147 individuals. There were no missing response items because the survey system required all answers to be complete. The questionnaire was approved by the Institutional Review Board (IRB).

### Study 2: Data collection

We collected data from May 31 to June 2, 2021 *via* the Qualtrics online panel service, which employed a quota sampling procedure to match the target race distribution of the U.S. national demographics.^1^ We used two methods to screen out low-quality responses: (1) the system does not record responses finished in less than half of the median time estimated from a preliminary test. The vendor thus provided us 272 completed submissions that fulfill the minimum time requirement. (2) Consistent with Study 1, we also excluded 14 participants reporting that they used social media more than 20 h a day. The quality check retained 258 valid responses. Since we set the survey system to require all answers to be complete, there were no missing values. The questionnaire design was approved by the Institutional Review Board (IRB).

### Study 2: A race-representative U.S. sample

The racial distribution of survey respondents in Study 2 was generally consistent with the racial distribution in the U.S. population (U.S. Census Bureau, 2021): White 72%, Black or African American 13%, Asian 5%, two or more races and other 10.00%. Our respondents were similar to the general adult population in terms of age (mean = 45.8 vs. 47 according to the U.S. Census Bureau estimate for the population over 18). Our sample contained more women (66%) than the total population (50.8%). They reported a median of 4 h of daily social media time (mean = 5.85, SD = 5.35), and 19 people (7.3%) reported zero hours of daily social media time. Only integer numbers are allowed for estimating social media use, so those who felt they did not use social media up to 1 h may choose to report 0.

### Measures

#### Anxiety

The dependent variable (of both studies) anxiety was measured by the Generalized Anxiety Disorder scale (Spitzer et al., 2006). This scale includes seven items asking about different anxiety symptoms in the past 2 weeks, and the respondent self-report the frequency of symptom occurrence, ranging from 0 (no occurrence in the past 2 weeks) to 3 (happening daily in the past 2 weeks). The measure is a validated metric in a variety of populations and is commonly used in research (Williams, 2014; Paez et al., 2020). In Study 1, both waves of the anxiety scale had high reliability (Cronbach’s alpha score of 0.90 at wave one and 0.90 at wave two). In Study 2, this scale also had high reliability (Cronbach’s alpha of 0.94).

#### Personal network size

A personal network is a social network from the perspective of the center person (Marin and Hampton, 2007). The size of one’s personal network thus often refers to the number of most close social contacts reported by the respondent (though other interpretations of the network are possible, such as a network made up of coworkers/classmates).

The survey instrument was adapted from extensive prior literature that has used personal network size as a proxy for perceived social support and demonstrated the reliability of the measure (Marin and Hampton, 2007). In public health research, this construct was shown to play a role in predicting a wide variety of health outcomes (Gardy et al., 2011; Preller et al., 2014; Marroquín et al., 2020; Tunçgenç et al., 2021). Supplementary Table S3 in Supplementary Material provides a summary of select prior applications. Personal network size was obtained by asking the respondent to identify a list of contacts that fit certain intimacy criteria as specified in the question. Following prior literature (see a list of prior applications in Supplementary Table S3), we asked, “think of the people you usually interact with within a typical month, by both online and offline communication methods. They could be family, friends and acquaintances or persons you feel close to.”

This question allowed the respondent to write down up to six social contacts’ names. Setting six as the upper limit followed many prior studies that measured the most intimate personal network size (see an expanded list of prior applications in Supplementary Table S3). This corresponds with the hierarchical social relations theory (Pollet et al., 2011), which suggests that the core layer of humans’ social network is typically no larger than five or six. Empirically, self-reported networks have been found to be similar in size with the naturally observed ego-networks on social media platforms (Haythornthwaite, 2000; Kim et al., 2007). Even though social networks are multidimensional, and it is hard to capture one’s full network with only one question, Marin and Hampton (2007) has empirically shown that an effective single-item question could reliably reflect the size of one’s network as obtained from a multiple-item questionnaire (*r* = 0.6 to 0.7).

In Study 1, 57.04% of the participants reported six contacts – the maximum number allowed, and 61.24% of the participants in Study 2 reported six contacts. This means most of the variance observed in this measure stems from the fact that nearly half of the participants nominated less than six names. The detailed distribution of this variable is shown in Supplementary Table S1 (Study 1) and Supplementary Table S2 (Study 2).

#### Social media use

We assessed time spent on social media in total and on news-related activities. Social media, in this study, is defined as web-based services that allow individuals to construct a public or semi-public user profile, connect with a list of users, and exchange information with others within the system (Boyd and Ellison, 2007). To help narrow down the concept of “social media,” participants were instructed to think of their behaviors on a list of mainstream social media platforms. The list is based on a 2019 Pew social media use report (Pew Research Center, 2019) and our interviews with Chinese, Japanese, and South Korean first-generation immigrants about the popular digital platforms in these countries. The final list includes YouTube, Facebook, Instagram, Pinterest, Snapchat, Twitter, WhatsApp, Reddit, WeChat, Kakao Friends, and Line. Note that social media is a subset of digital communication platforms, and we did not cover emails, live streaming platforms, Facetime, or Zoom – which are critical tools in our digital life but were not considered as typical social media.

First, we asked participants to estimate the total time per day spent on social media, which instrument has been widely used (Shensa et al., 2017; Paez et al., 2020). The question asked, “on a typical day, about how many hours do you spend using social media?” Participants were provided with open-ended boxes to write down a number to indicate daily hours. The distribution of this variable can be found in Supplementary Figure S1 (both studies).

Also, we differentiated news use from general use, because news use may be particularly problematic in the context of COVID-19 isolation (Aslam et al., 2020). The question was adapted from Bessière et al. (2008), which identified news consumption as an important purpose of social media use. We asked, “on a typical day, about how many hours do you spend browsing news feed on social media?” Participants were similarly asked to write down a number to indicate hours spent. News here is defined as published materials reported in media outlets on recent topics (Lazer et al., 2018), and we make no assumption about the content of the news being correct or incorrect.

#### Perceived impact of COVID-19

This variable was added and tested as a moderator of social media’s impact on anxiety in Study 2. The variable was measured by the question “To what extent has the situation associated with COVID-19 affected the way you live your life?” on a 5-point scale (1 = “no impact at all” to 5 = “impacted my life a great deal”), following Tull et al. (2020) and Qu et al. (2021).

#### Control variables

In Study 1, three demographic variables were collected at the first-wave survey, including respondents’ *age*, *years stayed in the U.S*. and *English proficiency*. Additionally, *anxiety* level at the first-wave survey was entered into the analysis. In Study 2, the control variables included respondents’ *age* and *sex.*

## Study one results

### Main effects

The age the respondents ranged between 18 to 40 (*M* = 25.45, *Mode* = 23, SD = 3.99). Typically, they have stayed in the U.S. for around 3 years (*M* = 2.85, *Median* = 3, SD = 1.63). Self-identified men make up 47.29% of the responses and 52.70% are women. Table 1 provides descriptive statistics and correlations of key variables.

**Table 1:** Study 1 descriptives and correlation (*N* = 147).

Variable	Mean	SD	1	2	3	4	5	6	7	8
1. Anxiety (t2)	5.91	4.25								
2. Anxiety (t1)	6.23	4.60	0.47^***^							
3. Ego-network size	4.55	2.06	−0.13	−0.17^*^						
4. Social media use (t2)	5.14	3.35	0.24^***^	0.10	−0.13					
5. Social media use non-news (t2)	1.84	2.52	0.13	0.06	0.04	0.60^***^				
6. Social media use news-related (t2)	3.30	2.74	0.18^*^	0.07	−0.20^*^	0.67^***^	−0.19^*^			
7. Age (t1)	25.45	3.99	0.16	−0.05	−0.12	−0.07	−0.19^*^	0.08		
8. Years in U.S.(t1)	2.85	1.63	0.16	0.02	−0.03	−0.16	−0.16	0.00	0.34^***^	
9. English proficiency (t1)	3.46	0.96	0.04	0.09	0.04	−0.23^**^	−0.21^**^	−0.08	0.23^**^	0.50^***^

* *p *< 0.05;

** *p *< 0.01; and

*** *p* < 0.00.

**Figure 1:**
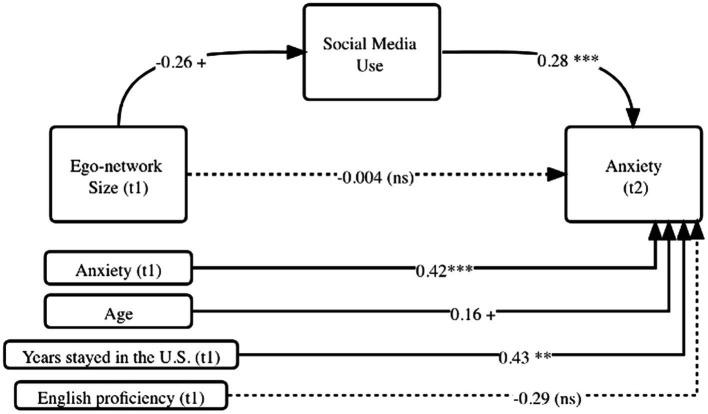
Model test results: personal network size, social media use and anxiety (Study 1). ^+^*p* < .0.10, ^**^p < 0.05, ^***^p < 0.01. Coefficients are unstandardized.

Path analysis with bootstrapped estimation of standard errors was conducted. To accurately capture the temporal changes in participants’ mental conditions, we also included the lagged dependent variable, as recommended in Bessière et al. (2010). Figure 1 shows the results of the mediation models.

To answer RQ1, we found that the size of one’s support network has no direct relationship with anxiety. After taking into account the mediation effect of social media, a larger support network size at a prior time did not predict anxiety in the future (*b* = −0.004, *ns*).

Regarding RQ2, the results showed that personal network size decreased the amount of time spent on social media in the next time period at a marginally significant level (*b* = −0.26, *p* < 0.1). That is, people who had a larger network at a prior time used social media less often in the future.

Analysis for RQ3 showed that increased social media use could lead to increased anxiety (*b* = 0.28, *p* < 0.01). Additionally, we tested for the indirect path effect (the impact of personal network size on anxiety through social media use) using the bootstrapped estimate approach as suggested in Hayes (2009). The indirect path coefficient estimate was −0.07 (unstandardized, 95% CI = −0.20 to −0.05), showing a significant mediation effect. These results demonstrated that social media use fully mediated the effect of personal network size on anxiety. The overall model for anxiety (t2) had an *R*^2^ value of 33.5%. The baseline linear model predicting anxiety with network size and control variables had an *R*^2^ value of 27.39%. The addition of social media use as a mediator in the model resulted in a $\Delta R^{2}$ = 6.11%.

### Differential effects of social media use types

The mean social media usage time was 5.14 h daily (SD = 3.35). The survey included an item asking respondents about their time spent on news consumption on social media. The current sample self-reported to spend an average of 3.3 h a day on news consumption (SD = 2.74). Social media use for non-news purpose was then calculated as the total hours of social media use minus the hours spent on news consumption (*M* = 1.84, SD = 2.52). A note of caution for interpreting these numbers is that the measure of non-news related social media use may be a conservative one. It is possible that people read their friends’ posts when they consume news on social media given the nature of the technology, and this mixed social media use is not covered by our measures. However, this should not hurt our results regarding the main effect of news-related social media use since our measure directly gauged the hours people spent on reading news on these platforms.

**Figure 2:**
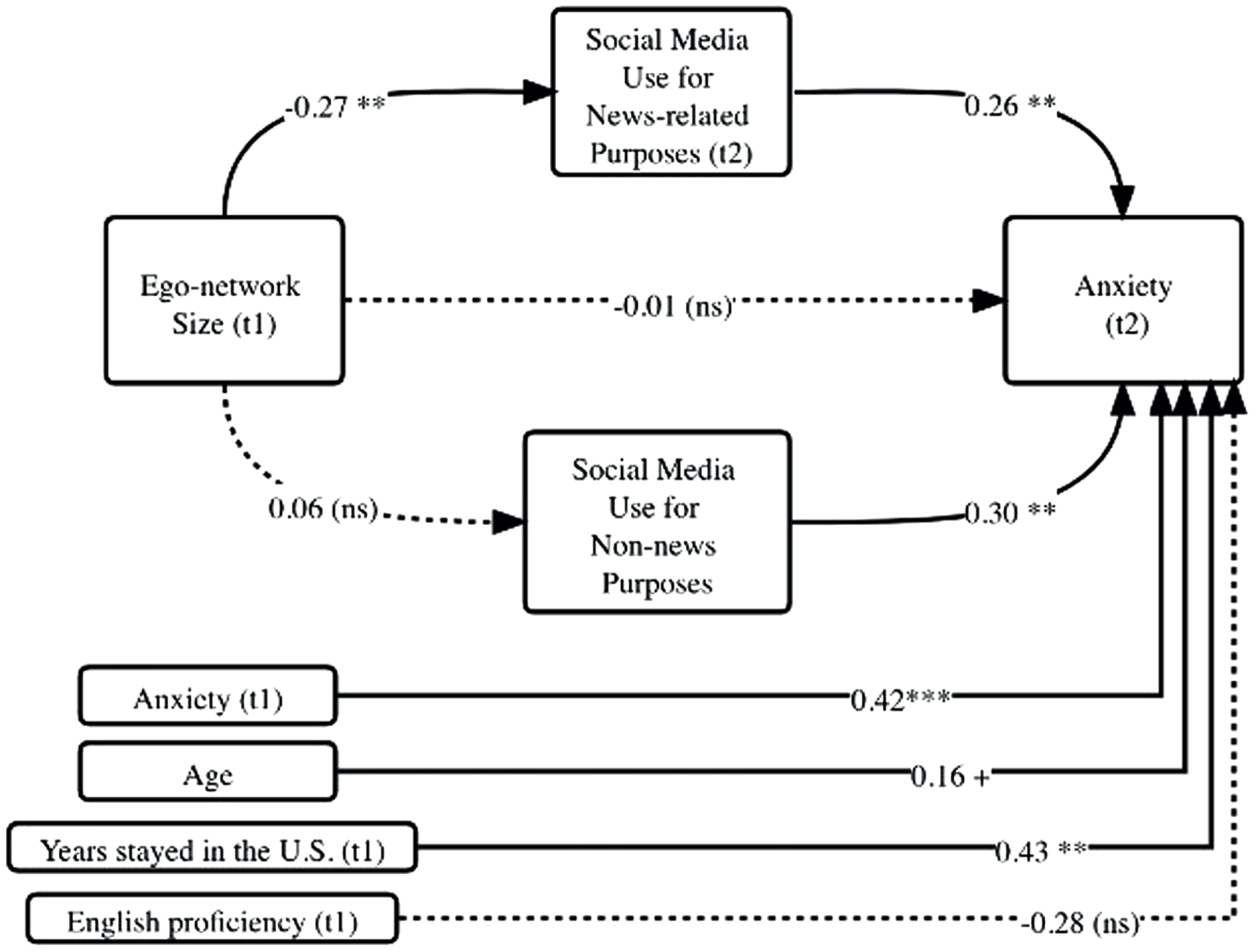
Path analysis results with two mediators: social media use for news-related and non-news purposes (Study 1). Dotted lines represent non-significant paths. ^+^*p* <0.10, ^**^p < 0.05, ^***^p < 0.01. Coefficients are unstandardized.

To address RQ4, both news and non-news social media use were entered as mediators and the proposed model was re-tested. Figure 2 illustrates the model test results. The path analysis revealed that only social media use for news consumption remained a significant mediator between personal network size and anxiety. As shown in Figure 2, the path from personal network size to news-related social media use was negative and significant (*b* = −0.27, *p* < 0.05). The path from news-related social media use to anxiety (*b* = 0.26, *p* < 0.05) remained positive and significant. Bootstrapped estimate of the indirect effect *via* news-related social media use (unstandardized estimate = −0.07, 95% CI = −0.22 to −0.014) confirmed this indirect effect. Hence, news-related social media use (but not non-news related social media use) mediated the relationship between personal network size and anxiety.

Together, the analysis further confirmed that time spent on news-related social media is the key variable that explains the relationship between social network size and anxiety. This observation did not apply to social media use for non-news purposes. The overall model for anxiety (t2) had an *R*^2^ value of 31.7%. Compared to the baseline linear model with network size as the predictor and the control variables, differentiating the two types of social media use in the mediation model led to a $\Delta R^{2}$ of 4.31%.

### Robustness check

#### Cross-lagged panel model

**Figure 3:**
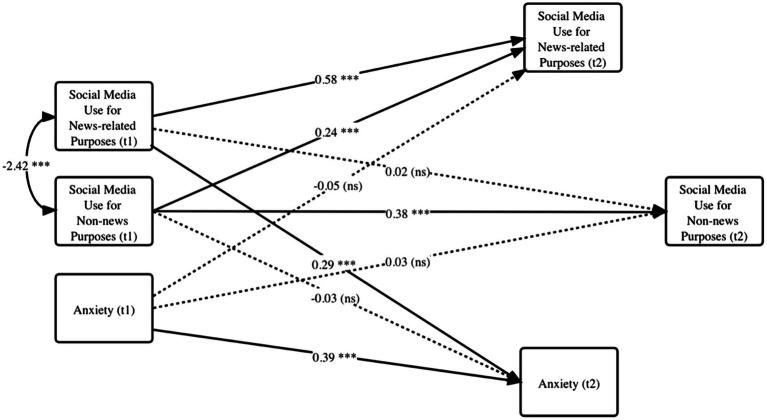
Cross-lagged panel model between social media use and anxiety at two time points (Study 1). Dotted lines represent non-significant paths. ^+^*p* < 0.10, ^**^p < 0.05, ^***^p < 0.01. Coefficients are unstandardized.

Since we employed social media use at Time 2 to predict anxiety level at the same time, one may question whether the relationship between the two variables was correlational. As a robustness check, we conducted a cross-lagged panel model to validate this relationship (Figure 3). In the hypothesized model, all variables at Time 2 were predicted by their initial value at Time 1 and by the value of the respective independent variable at Time 1. Additionally, the covariance between social media use for news and non-news purposes were allowed at the same time period.

The cross-lagged model confirmed the main model because the path from the initial anxiety level at Time 1 did not significantly predict social media use at Time 2 (neither news-related nor non-news related), but news-related social media use at Time 1 positively predicted anxiety at time 2 (*b* = 0.29, *p* < 0.01). The model fitted the data well, with χ2/*df* = 1.90, *p* > 0.1, CFI = 0.98, GFI = 0.98, RMSEA = 0.07, SRMR = 0.05, NFI = 0.97. This model confirmed the directional relationship from social media use to anxiety, rather than the other way around.

#### Step Heckman model for selection bias

Another potential concern of our analysis is the unit nonresponse error, as we employed a longitudinal design where respondents can voluntarily follow through or drop out from the second-wave survey. If the respondents self-selected themselves into the second wave on the basis of some endogenous attributes and such variables were correlated with anxiety or social media use, our estimates would suffer from selection bias. We tested for this selection bias using a 2-step Heckman selection model. This model tested for the assumption that the error of the selection function (first step model) and the error of the outcome function (second step model) are correlated. A significant test statistic would suggest the presence of selection bias. The analysis reported an inverse Mill’s ratio of-10.28 (*t* = 0.33, *p* = 0.71). The non-significant inverse Mill’s ratio indicates that no selection bias was detected by this test.

### Study two results

The descriptive statistics and correlations are presented in Table 2.

**Table 2:** Study 2 descriptives and correlation (*N* = 258).

Variable	Mean	SD	1	2	3	4	5
1. Anxiety	7.92	6.26					
2. Ego-network size	4.81	1.74	−0.07				
3. Social media use news-related	4.66	5.05	0.30^***^	−0.17^**^			
4. Social media use non-news	1.19	3.19	0.07	0.12^*^	−0.22^***^		
5. Perceived COVID-19 impact	3.78	1.04	0.01	0.13^*^	−0.09	0.08	
6. Age	45.82	17.20	−0.53^***^	0.07	−0.44^***^	−0.13^*^	0.14^*^

* *p* < 0.05;

** *p* < 0.01; and

*** *p* < 0.001.

**Figure 4:**
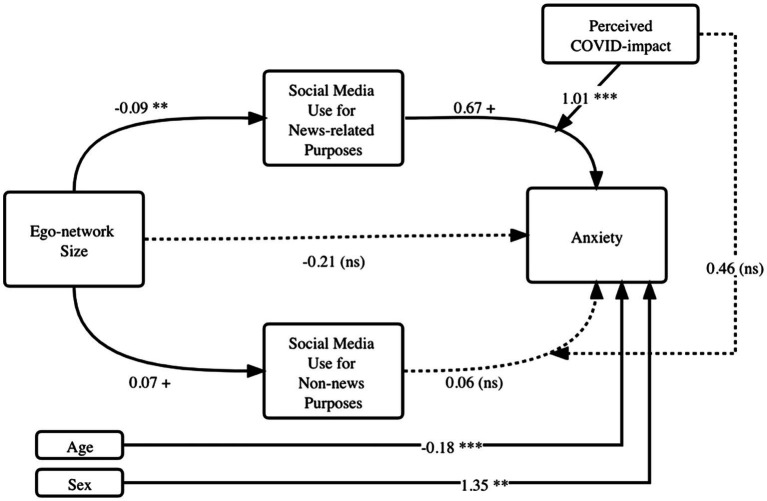
Moderated mediation analysis results (Study 2). Dotted lines represent non-significant paths. ^+^*p* < 0.10, ^**^p < 0.05, ^***^p < 0.01. Coefficients are unstandardized.

A moderated mediation model was analyzed to replicate the findings of Study 1 and to also test for the newly added moderator of perceived COVID-19 impact. Mediators and the moderator were mean-centered before entering into the model as suggested for moderation analysis (Dalal and Zickar, 2012). Figure 4 presents the results.

Consistent with Study 1, personal network size decreased the amount of time spent on news-related social media use (*b* = −0.09 *p* < 0.05). That is, people who had a smaller network tend to use social media more often for news-related purposes. Increased social media use for news could lead to increased anxiety (*b* = 0.67, *p <* 0.1).

Regarding the newly added moderator, perceived COVID-19 impact positively interacted with news-related social media use (*b* = 1.01, *p* < 0.01), which demonstrated that news-related social media use may be particularly harmful to those who feel they are impacted by COVID-19. To test for the index of mediated moderation for this path (from network size to news-related social media use to anxiety, and moderated by perceived COVID-impact), we calculated the product of the following paths’ coefficients: Coefficient from network size to news-related social media use ^*^ Coefficient of the interaction term (of news-related social media use and perceived COVID-impact). The bootstrapped confidence interval of this index did not include zero (*b* = −0.10, 95% CI = −0.25 to −0.02) which confirmed the moderated mediation effect. The moderated mediation relationship did not apply to social media use for non-news purposes (95% CI contains zero). The overall model for anxiety had an *R*^2^ value of 30.7%. Compared to the baseline linear model predicting anxiety with network size and control variables, this moderated mediation model increased the overall explanatory power ($\Delta R^{2}=1.3\%$). These results corroborated findings in Study 1.

### Summary of results

Table 3 summarizes the key findings, addressing each research question.

**Table 3:** Summary of key findings.

	• Study 1 finding	• Study 2 finding
• RQ1 (personal network and anxiety)	• No direct effect	• No direct effect
• RQ2 (personal network and social media use)	• Larger personal network size →less social media use	• Larger personal network size →less social media use
• RQ3 (social media use and anxiety)	• Social media use → higher levels of anxiety	• Social media use →higher levels of anxiety
• RQ4 (differential effects of news vs. non-news social media)	• Social media for news →higher levels of anxiety; • Social media for non-news →higher levels of anxiety	• Social media for news →higher levels of anxiety
• Additional moderator of perceived COVID-19 impact	• NA	• Perceived COVID-19 impact positively moderates the link between social media for news and anxiety

## Discussion

### Principal findings

Adopting the social network perspective, we conducted two surveys to investigate how personal network and social media use are related to anxiety during a public health crisis. Overall, more social media use time predicted higher levels of anxiety. Second, a large network decreased time spent on using social media (especially using social media for news), which then reduced anxiety (see Figures 2, 3). In this sense, a strong social support network provides mental “protection” by distracting us away from potentially disturbing news on social media. Amid large-scale social lockdown, this finding reminds us of the critical supporting role that our closest social circle plays. Third, consuming news on social media resulted in increased anxiety, which was amplified for individuals already feeling the impact of COVID-19 (see Figure 4). Social media today has become a primary source for information consumption (Geirdal et al., 2021). These findings warn us of a situation where those who already feel impacted by the pandemic may experience more anxiety when they consume news on social media. It confirmed prior findings that researchers should be cautious of *how much* people consume news amid a global pandemic (Aslam et al., 2020).

### Social media’s negative impact on mental health

The findings of the two studies were consistent in that social media use was associated with heightened anxiety. This conclusion was consistent with recent correlational studies (Boursier et al., 2020; Cauberghe et al., 2021; Geirdal et al., 2021), but our study provided more robust evidence because of the unique research design. Most studies examining social media and mental illness during the pandemic adopted a cross-sectional design, and understandably so – scholars needed to first have a quick assessment of the prevalence of mental conditions during the pandemic across a variety of populations and establish a correlational relationship. This article extended prior studies by adding a longitudinal design, and a comparative replication study. Total social media use predicted higher anxiety levels in the longitudinal survey of East Asian international students studying in the U.S. (Study 1) and a cross-sectional, race-representative sample of U.S. adults (Study 2). The two samples and data collection periods were highly distinct, so the consistent findings were quite robust. Study 1 focuses on a subpopulation that may be suffering from a series of social and political stressors during the earlier months of the outbreak, while Study 2 replicated and extended Study 1 with a more representative sample after the pandemic had developed for nearly a year and when half of the U.S. population has been vaccinated.

It also tested the possibility of the bidirectional causal relationship between social media use and anxiety. The robustness check using a cross-lagged effect model helped us rule out an alternative explanation—it may be anxiety (a personal predisposing factor) leading to more social media use. With the two-wave data, we found that it was social media use predicting higher anxiety, but not the other way around. However, it is possible that the two variables reinforce one another if observed in the long term. A limitation of the two-wave survey design is that we were unable to fully explicate causal relationships beyond a single time interval. A three-wave panel study before the pandemic (Tandoc and Goh, 2021) found that Facebook use at time 1 predicted increased depression level at time 2, and depression level at time 2 intensified Facebook use at time 3. Our finding was consistent with their reported first path (time 1 to time 2), but we did not have data to further untangle the intricate dynamics between social media and mental health over longer time spans.

### Debate about personal network and social media use

Addressing the debate about the relationship between personal network and social media use, we found support for the trade-off effect (larger social network leading to less social media use) instead of the augmentation effect. In fact, the “trade-off” effect observed here does not mean that family-and-friends time competes with new-friends time. Two different samples both revealed that the time and energy spent on maintaining close relationships only reduced the amount of time on consuming social media news, and not for other purposes.

Thus, this article points to a new interpretation of the “trade-off effect” between personal network and the necessity of information-seeking on social media. An individual with stronger and more fulfilling relationships may feel less anxious to access news on social media, which is the primary purpose of social media use reported by our respondents. The theory of incidental news exposure (Lee and Kim, 2017) offers a possible explanation, positing that social networks can serve as a source of incidental information, thus reducing the necessity of checking the news online. In addition, the conditional effect of perceived COVID-19 impact supports the idea of COVID Stress Syndrome (Taylor et al., 2020), which argued that news-checking and reassurance-seeking behavior is a strategy to cope with COVID stress. When people obtained enough social support to ease COVID stress, the need for news-checking may decrease.

### Types of social media use and differential effects on mental health

Moreover, the present study addressed the call for more careful considerations about the types of technology use and their differential consequences (Kingsbury et al., 2021). It also helped explain the mixed findings regarding the effects of social media use on mental well-being. Our analysis showed that social media use for news, compared to non-news use, consistently predicted higher levels of anxiety in both studies. The results hold after considering the possible bidirectional relationships using a cross-lagged effect model.

While prior literature conducted in the pre-pandemic situation mostly focused on differentiating between passive and active social media use (Wang et al., 2018; Yuen et al., 2019; Kingsbury et al., 2021), not enough attention has been paid to social media use specifically for news purposes and its mental health impact. In the context of COVID-19, the “information-seeking” affordance of social media becomes a crucial aspect highlighted in many empirical observations (Bento et al., 2020; Stainback et al., 2020; Galido et al., 2021). Literature provided at least two theoretical explanations regarding this negative link –– due to the context of a crisis, or due to the proliferation of misinformation that overwhelmed viewers.

On the one hand, it is reasonable that news-related activities during crises, in general, predict negative mental outcomes. This conclusion has been validated multiple times in contexts of major disasters such as exposure to the 9/11 attack and the Iraq War (Silver et al., 2013), 2013 Boston Marathon bombings (Holman et al., 2014) and the 2016 Orlando Pulse nightclub massacre (Thompson et al., 2019). This article provided another test of the negative impact of news exposure during times of crises, and more importantly, it focused on social media-based news use, instead of a combined news exposure (that is not channel-specific) like the above-mentioned studies.

On the other hand, we speculate that the proliferation of misinformation during the pandemic, which some even named as an “infodemic” beyond the disease pandemic, also leads to undesirable consequences. Studies on COVID-19 have shown that misinformation was more frequently tweeted than science-based evidence or legit public health recommendations (Pulido et al., 2020), and it could demotivate information seeking and thoughtful processing of COVID-19 information (Kim et al., 2020). However, our data do not contain information regarding misinformation consumption *via* social media, which is a critical direction for future research.

Admittedly, this finding should be interpreted with caution because social media is a multitude of activities involving complicated psychological processes (Hyun and Kim, 2015). A simple binary distinction between news and non-news uses is not exhaustive. Literature has suggested several variables related to social media news use that is worthy of future investigations: the content of news (i.e., whether it is a description of the crisis or not), the different modes of news consumption behaviors (i.e., such as news reception, news following, and news dissemination), the motivation of news consumption (i.e., whether it is driven by fear or driven by a need for knowledge), the legitimacy of the information (i.e., true content versus misinformation/disinformation), and the audience’s processing strategy of negative news (i.e., whether one denies or accepts the information). We also acknowledge that the current study did not cover online activities on channels that are not defined as social media (such as Zoom, Facetime, live streaming sites, or even emails), despite their importance during the pandemic. Studying some or all of the activities on the digital technology applications discussed above will lead to fruitful results in the future. The current project joins many other such efforts to understand this highly complex process. We believe it provides some initial evidence that social media should be considered as a primary source of information consumption.

### Limitations and future directions

This study has several limitations. First, even though our measure of network size was shown to be reliable (Marin and Hampton, 2007), self-reported social network size was a proxy of “perceived” social support instead of “received” social support. Perceived and received social support have been conceptualized as different variables. We chose a measure of perceived social support because perceived social support, rather than the received one, was found to be the primary factor that influences health outcomes (Haber et al., 2007). Nevertheless, network size only emphasized one quantitative aspect of one’s social network and may not fully represent one’s ability to accrue social support or the quality of the support one received. We also did not have information about the structure of one’s personal network, such as density, centrality, tie strength, or subgroups, which could provide valuable insights into one’s interpersonal environment. Future work can examine these network variables and ask for more contextual information such as the perceived quality and amount of support received.

Likewise, we relied on self-reported measures of social media usage. Whereas we adopted this measure from prior studies examining social media use (Hill and Zheng, 2018; Paez et al., 2020), we acknowledge that it is subject to personal perceptions, recall error, and may be primed by one’s more recent social media usage. Future studies can combine behavioral trace data that objectively record online social interactions with self-reported data to explicate these relationships further.

Third, the unique research context – a major public health crisis – limited the findings’ generalizability. It could be that the effect is moderated by the occurrence of public crises such as natural disasters or political upheavals, during which social media is likely to facilitate the spread of negative sentiments (Aslam et al., 2020).

Fourth, the positive interaction effect between news-related social media use and the perceived impact of COVID-19 was merely a correlational connection, instead of a causal one. We cannot determine if it is COVID-19 stress caused more news consumption and reassurance-seeking or the other way around. This issue could be best resolved by collecting three or more waves of longitudinal data, or even conducting randomized control trials.

Fifth, the sample sizes were relatively small, compared to representative national surveys. This could limit the generalizability of the current findings.

Lastly, we collected online panel data obtained from Qualtrics and used pre-determined quotas to match U.S. population race distribution. This decision serves the purpose of this article well, because we were primarily interested in people who had social media access (Walter et al., 2016). However, we acknowledge that there are documented limitations of such panels, such as respondents’ pre-existing Internet access and that participants’ willingness to opt-in paid research panels, which may bias the results.

## Conclusion

Two studies across different subpopulations consistently found that more social media use was associated with increased anxiety. Personal network is a critical social antecedent of social media use. Maintaining a strong social support circle will protect us by lowering the time spent on social media and thus lowering anxiety. Additionally, social media’s negative role is mainly explained by news-related activities on social media, and the strength of this relationship is conditioned by one’s perception of COVID-19 impact. Together, these results demonstrate the increasingly critical and multifaceted role of communication technologies in affecting mental health conditions.

## Data availability statement

The raw data supporting the conclusions of this article will be made available by the authors, without undue reservation.

## Ethics statement

The studies involving human participants were reviewed and approved by University of Southern California Institutional Review Board. The patients/participants provided their written informed consent to participate in this study.

## Author contributions

RR contributed to conceptualization, methodology, data investigation, writing of the manuscript, visualization, and funding acquisition. BY contributed to conceptualization, data investigation, and writing of the manuscript. All authors contributed to the article and approved the submitted version.

## Funding

This research was supported by China Ministry of Education Humanity and Social Science Research Youth Program (#21YJC860018), Shanghai Pujiang Talents Program Category C (#21PJC079), and SJTU - International Association of Cultural and Creative Industry Research funding program. The first author also received funding from USC Annenberg School’s Kaleigh Finnie Memorial Endowment while studying as a PhD student.

## Conflict of interest

The authors declare that the research was conducted in the absence of any commercial or financial relationships that could be construed as a potential conflict of interest.

## Publisher’s note

All claims expressed in this article are solely those of the authors and do not necessarily represent those of their affiliated organizations, or those of the publisher, the editors and the reviewers. Any product that may be evaluated in this article, or claim that may be made by its manufacturer, is not guaranteed or endorsed by the publisher.

## Footnotes

^1^For more information about Qualtrics’ s quota sampling procedure, visit: https://www.qualtrics.com/support/survey-platform/survey-module/survey-tools/quotas/
