# Supplementary material for: Personal network protects, social media harms: Evidence from two surveys during the COVID-19 pandemic
Source: Front Psychol. 2022 Aug 22;13:964994. doi: 10.3389/fpsyg.2022.964994 (PMC9441876; doi:10.3389/fpsyg.2022.964994)
Supplement: Supplementary file 1 [file Data_Sheet_1.docx]

Supplementary Material

# Supplementary Figure S1

Distribution Histogram of Daily Hours of Social Media Use


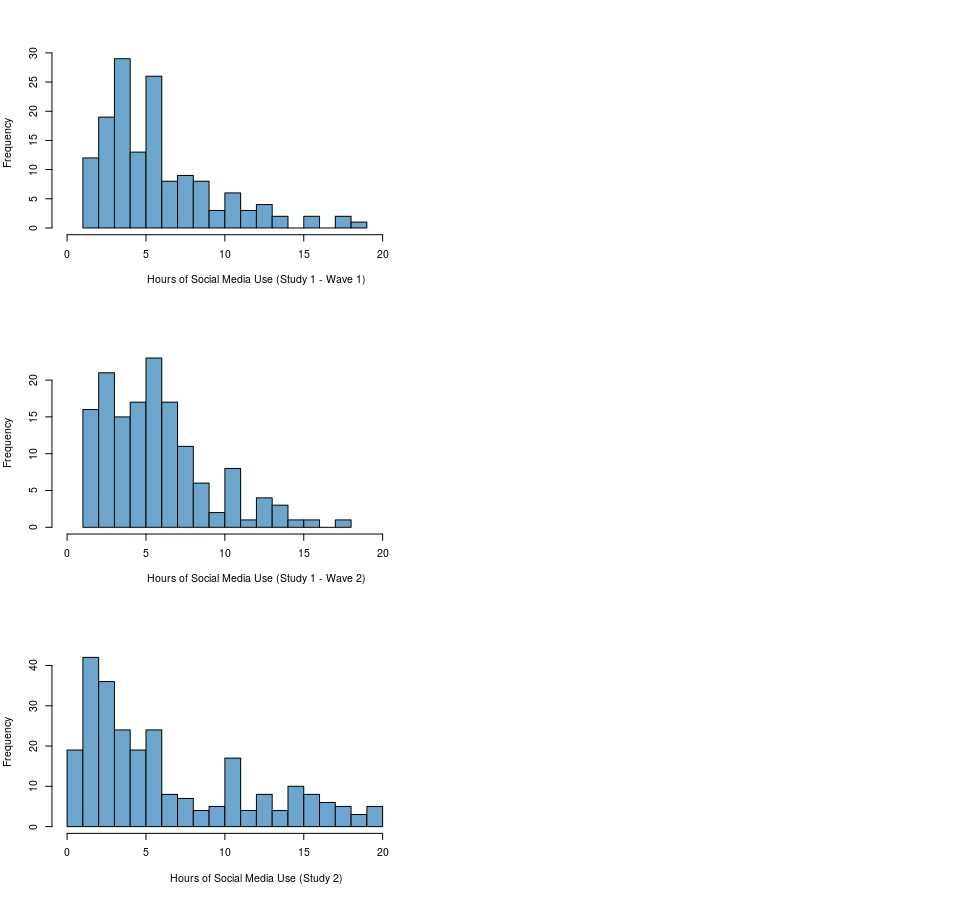


# Supplementary Table S1

Distribution of Ego-network Size (Study 1)

| Network size | Frequency | Relative Percentage |
| --- | --- | --- |
| 0 | 15 | 10.20% |
| 1 | 5 | 3.40% |
| 2 | 6 | 4.08% |
| 3 | 11 | 7.48% |
| 4 | 15 | 10.20% |
| 5 | 11 | 7.48% |
| 6 | 84 | 57.14% |
|  | N = 147 |  |

# Supplementary Table S2

Distribution of Ego-network Size (Study 2)

| Network size | Frequency | Relative Percentage |
| --- | --- | --- |
| 0 | 5 | 1.93% |
| 1 | 15 | 5.81% |
| 2 | 15 | 5.81% |
| 3 | 26 | 10.07% |
| 4 | 23 | 8.91% |
| 5 | 16 | 6.20% |
| 6 | 158 | 61.24% |
|  | N = 258 |  |

# Measuring Personal Network Size

The measurement of personal network size a widely applied technique in social network research to obtain information about people’s personal network. Table S3 provides a comparison of exemplary applications used in 11 prior studies, ranging from sociology to public health, conducted from 1980s to the recent COVID-19 period. Depending on their research questions, researchers can be quite flexible in specifying the nature of the social connections that they are interested in, the upper limit of the network size, and the time frame to recall the network. Some scholars also chose to explore a multitude of personal networks by soliciting social relations in different contexts. From Table S3, it is clear that although the wordings of name generators vary, intimacy or closeness is a common choice of the criterion for inclusion of a contact. Moreover, setting the upper limit of network size to five or six is a common practice. Even when an upper bound was not specified to facilitate participants’ recall, the average network size reported by the subjects are often below 5. These scholarly practices guided the survey instrument used in the current research.

**Table S3.** A Comparison of Name Generators used in Literature

| Reference | Nature of Connection | Numerical Limit | Time Frame | Mean size | Sd |
| --- | --- | --- | --- | --- | --- |
| Toronto East York Survey (Wellman, 1979) | Persons (outside your home) that you feel closest to | 6 | No | 4.58 | - |
| Northern California Communities Study (Fischer, 1982) | Nine different types of social exchanges including looking after their homes, talking about jobs, social hangouts, etc. | 8 each | No | - | - |
| General Social Survey  (Burt, 1984) | Persons with whom the respondent discussed personal matters | 5 | Last six months | 3^a^ | - |
| British Columbia TB Outbreak Study (Gardy et al., 2011) | Your closest family members; your closet friends or the people you hung out with the most | No | Last year | - | - |
| Georgia Assisted Living Residents Study (Perkins et al., 2013) | Three levels of questions:  Individuals to whom they felt so close that it was hard to imagine life without (inner circle); to whom they felt still felt close but not as important as those placed in the inner circle (middle circle); people not yet mentioned but who were still important enough (outer circle) | Three circles combined maximum 20 | No | 4.83 (inner circle) | 2.94 |
| Zurich Cocaine Cognition Study (Preller et al., 2014) | Six separate questions:  Persons in the areas of household, family, work or apprenticeship, friends, neighbors, and others, respectively | No | Last four weeks | - | - |
| UCNets Study of Younger and Older Adults in SF Bay Area (Child & Lawton, 2020) | Persons that the respondent feels close to | No | No | 4.37 (younger adults sample) | 4.54 |
|  |  |  |  | 4.58  (older adults sample) | 4.31 |
| A U.S. COVID-19 Social Distancing Behavior Study (Marroquín et al., 2020) | Persons talked to in 12 different social capacities (including partners, parents, children, coworkers, classmates, neighbors, group members, etc.) | No | Last two weeks | 13.67 (12 social capacities combined)^b^ | 8.71 |
| A Global COVID-19 Prevention Adherence Study (Tunçgenç et al., 2021) | People they had voluntarily had a conversation with and would turn to for advice or comfort | 20 | Last week | - | - |
| A U.S. COVID-19 Information Avoidance and Social Network Survey (Qu et al., 2021) | Three separate questions:  Persons with whom talked about COVID-19; who always turned to me to discuss COVID-19; who were a burden to me because of COVID-19 related needs | 5 each | No | 4.13 | 2.51 |

*Note*. a. This result is obtained in the General Social Survey administered in 1985 – one year after the Burt (1984) paper which proposed this measure.

b. This number is high because it is not a measure of a single network, but a count sum based on 12 different social networks. This measure also did not differentiate between different levels of relational closeness. Example questions were “How many fellow students or teachers do you talk to at least once every two weeks?” and “How many neighbors do you talk to …”. The closest social circle size is not explicitly provided.

- indicates that number was not provided in the article.

*For comparison, in the current Study 1, mean network size is 4.5 ± 2.07, and the mean network size in Study 2 is 4.83±1.74.
